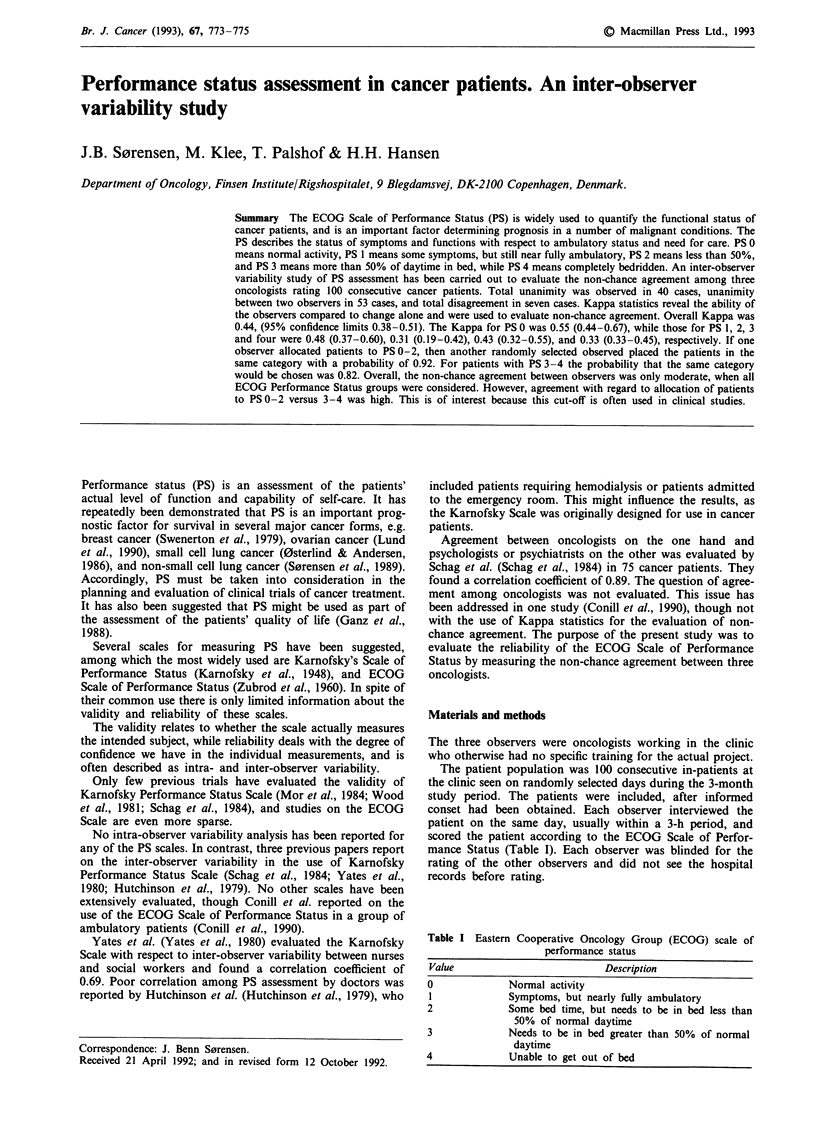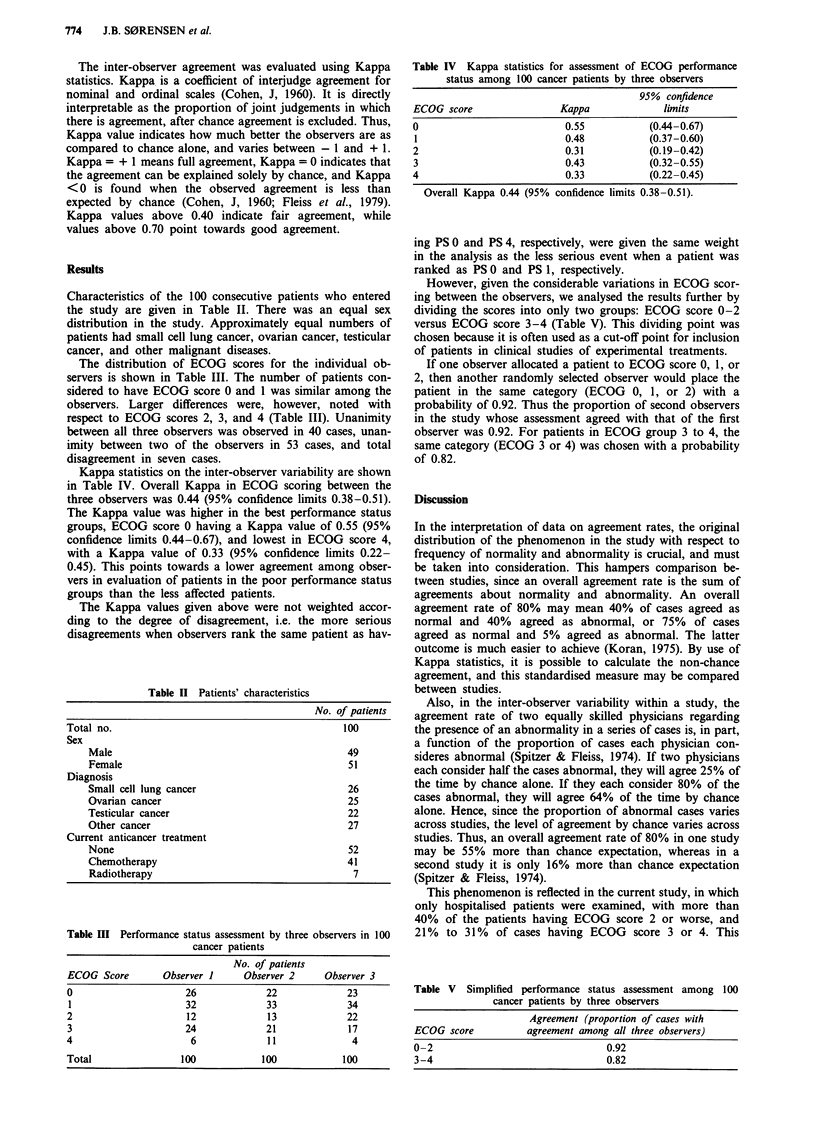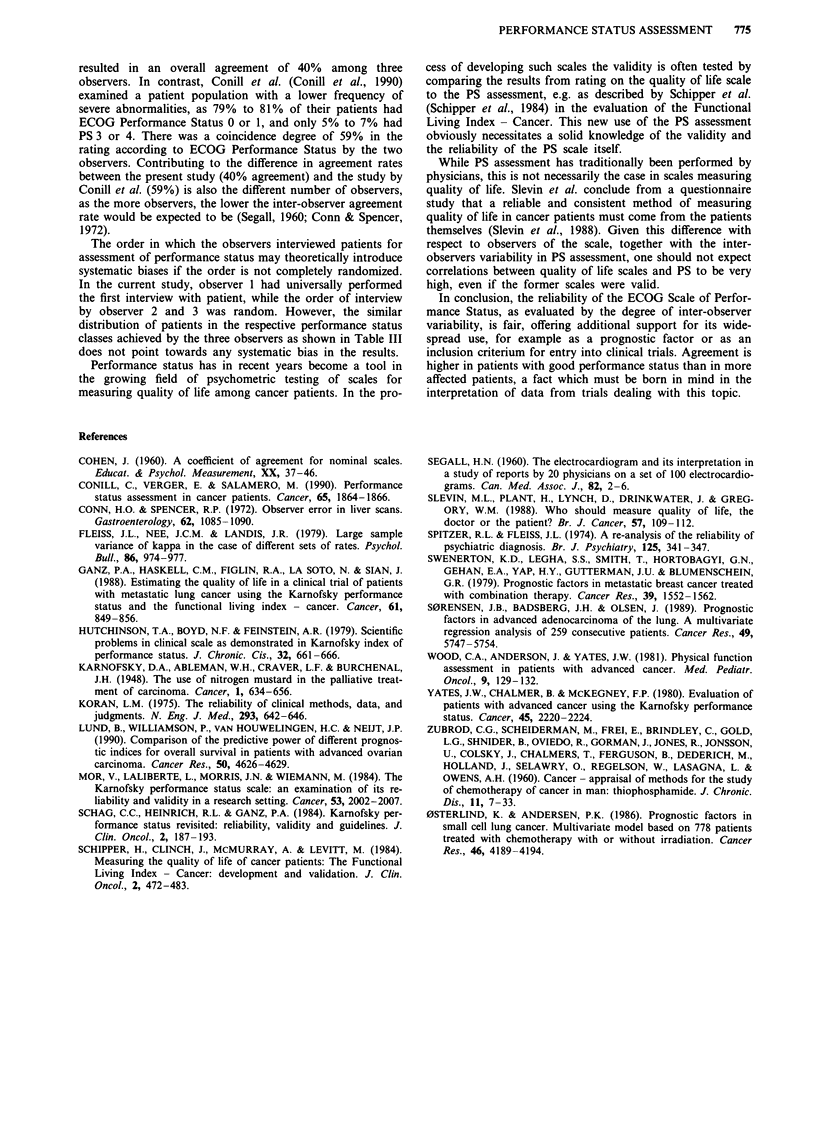# Performance status assessment in cancer patients. An inter-observer variability study.

**DOI:** 10.1038/bjc.1993.140

**Published:** 1993-04

**Authors:** J. B. Sørensen, M. Klee, T. Palshof, H. H. Hansen

**Affiliations:** Department of Oncology, Finsen Institute/Rigshospitalet, Copenhagen, Denmark.

## Abstract

The ECOG Scale of Performance Status (PS) is widely used to quantify the functional status of cancer patients, and is an important factor determining prognosis in a number of malignant conditions. The PS describes the status of symptoms and functions with respect to ambulatory status and need for care. PS 0 means normal activity, PS 1 means some symptoms, but still near fully ambulatory, PS 2 means less than 50%, and PS 3 means more than 50% of daytime in bed, while PS 4 means completely bedridden. An inter-observer variability study of PS assessment has been carried out to evaluate the non-chance agreement among three oncologists rating 100 consecutive cancer patients. Total unanimity was observed in 40 cases, unanimity between two observers in 53 cases, and total disagreement in seven cases. Kappa statistics reveal the ability of the observers compared to change alone and were used to evaluate non-chance agreement. Overall Kappa was 0.44, (95% confidence limits 0.38-0.51). The Kappa for PS 0 was 0.55 (0.44-0.67), while those for PS 1, 2, 3 and four were 0.48 (0.37-0.60), 0.31 (0.19-0.42), 0.43 (0.32-0.55), and 0.33 (0.33-0.45), respectively. If one observer allocated patients to PS 0-2, then another randomly selected observed placed the patients in the same category with a probability of 0.92. For patients with PS 3-4 the probability that the same category would be chosen was 0.82. Overall, the non-chance agreement between observers was only moderate, when all ECOG Performance Status groups were considered. However, agreement with regard to allocation of patients to PS 0-2 versus 3-4 was high. This is of interest because this cut-off is often used in clinical studies.


Br. J. Cancer (1993), 67, 773–775 © Macmillan Press Ltd., 1993

### Performance status assessment in cancer patients. An inter-observer variability study

J.B. Sørensen, M. Klee, T. Palshof & H.H. Hansen

*Department of Oncology, Finsen Institute/Rigshospitalet, 9 Blegdamsvej, DK-2100 Copenhagen, Denmark.*

**Summary** The ECOG Scale of Performance Status (PS) is widely used to quantify the functional status of cancer patients, and is an important factor determining prognosis in a number of malignant conditions. The PS describes the status of symptoms and functions with respect to ambulatory status and need for care. PS 0 means normal activity, PS 1 means some symptoms, but still near fully ambulatory, PS 2 means less than 50%, and PS 3 means more than 50% of daytime in bed, while PS 4 means completely bedridden. An inter-observer variability study of PS assessment has been carried out to evaluate the non-chance agreement among three oncologists rating 100 consecutive cancer patients. Total unanimity was observed in 40 cases, unanimity between two observers in 53 cases, and total disagreement in seven cases. Kappa statistics reveal the ability of the observers compared to change alone and were used to evaluate non-chance agreement. Overall Kappa was 0.44, (95% confidence limits 0.38–0.51). The Kappa for PS 0 was 0.55 (0.44–0.67), while those for PS 1, 2, 3 and four were 0.48 (0.37–0.60), 0.31 (0.19–0.42), 0.43 (0.32–0.55), and 0.33 (0.33–0.45), respectively. If one observer allocated patients to PS 0–2, then another randomly selected observed placed the patients in the same category with a probability of 0.92. For patients with PS 3–4 the probability that the same category would be chosen was 0.82. Overall, the non-chance agreement between observers was only moderate, when all ECOG Performance Status groups were considered. However, agreement with regard to allocation of patients to PS 0–2 versus 3–4 was high. This is of interest because this cut-off is often used in clinical studies.

Performance status (PS) is an assessment of the patients' actual level of function and capability of self-care. It has repeatedly been demonstrated that PS is an important prognostic factor for survival in several major cancer forms, e.g. breast cancer (Swenerton *et al.*, 1979), ovarian cancer (Lund *et al.*, 1990), small cell lung cancer (Østerlind & Andersen, 1986), and non-small cell lung cancer (Sørensen *et al.*, 1989). Accordingly, PS must be taken into consideration in the planning and evaluation of clinical trials of cancer treatment. It has also been suggested that PS might be used as part of the assessment of the patients' quality of life (Ganz *et al.*, 1988).

Several scales for measuring PS have been suggested, among which the most widely used are Karnofsky's Scale of Performance Status (Karnofsky *et al.*, 1948), and ECOG Scale of Performance Status (Zubrod *et al.*, 1960). In spite of their common use there is only limited information about the validity and reliability of these scales.

The validity relates to whether the scale actually measures the intended subject, while reliability deals with the degree of confidence we have in the individual measurements, and is often described as intra- and inter-observer variability.

Only few previous trials have evaluated the validity of Karnofsky Performance Status Scale (Mor *et al.*, 1984; Wood *et al.*, 1981; Schag *et al.*, 1984), and studies on the ECOG Scale are even more sparse.

No intra-observer variability analysis has been reported for any of the PS scales. In contrast, three previous papers report on the inter-observer variability in the use of Karnofsky Performance Status Scale (Schag *et al.*, 1984; Yates *et al.*, 1980; Hutchinson *et al.*, 1979). No other scales have been extensively evaluated, though Conill *et al.* reported on the use of the ECOG Scale of Performance Status in a group of ambulatory patients (Conill *et al.*, 1990).

Yates *et al.* (Yates *et al.*, 1980) evaluated the Karnofsky Scale with respect to inter-observer variability between nurses and social workers and found a correlation coefficient of 0.69. Poor correlation among PS assessment by doctors was reported by Hutchinson *et al.* (Hutchinson *et al.*, 1979), who included patients requiring hemodialysis or patients admitted to the emergency room. This might influence the results, as the Karnofsky Scale was originally designed for use in cancer patients.

Agreement between oncologists on the one hand and psychologists or psychiatrists on the other was evaluated by Schag *et al.* (Schag *et al.*, 1984) in 75 cancer patients. They found a correlation coefficient of 0.89. The question of agreement among oncologists was not evaluated. This issue has been addressed in one study (Conill *et al.*, 1990), though not with the use of Kappa statistics for the evaluation of non-chance agreement. The purpose of the present study was to evaluate the reliability of the ECOG Scale of Performance Status by measuring the non-chance agreement between three oncologists.

### Materials and methods

The three observers were oncologists working in the clinic who otherwise had no specific training for the actual project.

The patient population was 100 consecutive in-patients at the clinic seen on randomly selected days during the 3-month study period. The patients were included, after informed conset had been obtained. Each observer interviewed the patient on the same day, usually within a 3-h period, and scored the patient according to the ECOG Scale of Performance Status (Table I). Each observer was blinded for the rating of the other observers and did not see the hospital records before rating.

**Table I** Eastern Cooperative Oncology Group (ECOG) scale of performance status

| *Value* | *Description* |
|---|---|
| 0 | Normal activity |
| 1 | Symptoms, but nearly fully ambulatory |
| 2 | Some bed time, but needs to be in bed less than 50% of normal daytime |
| 3 | Needs to be in bed greater than 50% of normal daytime |
| 4 | Unable to get out of bed |

Correspondence: J. Benn Sørensen.
Received 21 April 1992; and in revised form 12 October 1992.

The inter-observer agreement was evaluated using Kappa statistics. Kappa is a coefficient of interjudge agreement for nominal and ordinal scales (Cohen, J, 1960). It is directly interpretable as the proportion of joint judgements in which there is agreement, after chance agreement is excluded. Thus, Kappa value indicates how much better the observers are as compared to chance alone, and varies between − 1 and + 1. Kappa = + 1 means full agreement, Kappa = 0 indicates that the agreement can be explained solely by chance, and Kappa <0 is found when the observed agreement is less than expected by chance (Cohen, J, 1960; Fleiss et al., 1979). Kappa values above 0.40 indicate fair agreement, while values above 0.70 point towards good agreement.

### Results

Characteristics of the 100 consecutive patients who entered the study are given in Table II. There was an equal sex distribution in the study. Approximately equal numbers of patients had small cell lung cancer, ovarian cancer, testicular cancer, and other malignant diseases.

The distribution of ECOG scores for the individual observers is shown in Table III. The number of patients considered to have ECOG score 0 and 1 was similar among the observers. Larger differences were, however, noted with respect to ECOG scores 2, 3, and 4 (Table III). Unanimity between all three observers was observed in 40 cases, unanimity between two of the observers in 53 cases, and total disagreement in seven cases.

Kappa statistics on the inter-observer variability are shown in Table IV. Overall Kappa in ECOG scoring between the three observers was 0.44 (95% confidence limits 0.38–0.51). The Kappa value was higher in the best performance status groups, ECOG score 0 having a Kappa value of 0.55 (95% confidence limits 0.44–0.67), and lowest in ECOG score 4, with a Kappa value of 0.33 (95% confidence limits 0.22–0.45). This points towards a lower agreement among observers in evaluation of patients in the poor performance status groups than the less affected patients.

The Kappa values given above were not weighted according to the degree of disagreement, i.e. the more serious disagreements when observers rank the same patient as having PS 0 and PS 4, respectively, were given the same weight in the analysis as the less serious event when a patient was ranked as PS 0 and PS 1, respectively.

However, given the considerable variations in ECOG scoring between the observers, we analysed the results further by dividing the scores into only two groups: ECOG score 0–2 versus ECOG score 3–4 (Table V). This dividing point was chosen because it is often used as a cut-off point for inclusion of patients in clinical studies of experimental treatments.

If one observer allocated a patient to ECOG score 0, 1, or 2, then another randomly selected observer would place the patient in the same category (ECOG 0, 1, or 2) with a probability of 0.92. Thus the proportion of second observers in the study whose assessment agreed with that of the first observer was 0.92. For patients in ECOG group 3 to 4, the same category (ECOG 3 or 4) was chosen with a probability of 0.82.

**Table II** Patients' characteristics

| | *No. of patients* |
|---|---|
| Total no. | 100 |
| Sex | |
| Male | 49 |
| Female | 51 |
| Diagnosis | |
| Small cell lung cancer | 26 |
| Ovarian cancer | 25 |
| Testicular cancer | 22 |
| Other cancer | 27 |
| Current anticancer treatment | |
| None | 52 |
| Chemotherapy | 41 |
| Radiotherapy | 7 |

**Table III** Performance status assessment by three observers in 100 cancer patients

| *ECOG Score* | *No. of patients* Observer 1 | *Observer 2* | *Observer 3* |
|---|---|---|---|
| 0 | 26 | 22 | 23 |
| 1 | 32 | 33 | 34 |
| 2 | 12 | 13 | 22 |
| 3 | 24 | 21 | 17 |
| 4 | 6 | 11 | 4 |
| Total | 100 | 100 | 100 |

**Table IV** Kappa statistics for assessment of ECOG performance status among 100 cancer patients by three observers

| *ECOG score* | *Kappa* | *95% confidence limits* |
|---|---|---|
| 0 | 0.55 | (0.44–0.67) |
| 1 | 0.48 | (0.37–0.60) |
| 2 | 0.31 | (0.19–0.42) |
| 3 | 0.43 | (0.32–0.55) |
| 4 | 0.33 | (0.22–0.45) |

Overall Kappa 0.44 (95% confidence limits 0.38–0.51).

### Discussion

In the interpretation of data on agreement rates, the original distribution of the phenomenon in the study with respect to frequency of normality and abnormality is crucial, and must be taken into consideration. This hampers comparison between studies, since an overall agreement rate is the sum of agreements about normality and abnormality. An overall agreement rate of 80% may mean 40% of cases agreed as normal and 40% agreed as abnormal, or 75% of cases agreed as normal and 5% agreed as abnormal. The latter outcome is much easier to achieve (Koran, 1975). By use of Kappa statistics, it is possible to calculate the non-chance agreement, and this standardised measure may be compared between studies.

Also, in the inter-observer variability within a study, the agreement rate of two equally skilled physicians regarding the presence of an abnormality in a series of cases is, in part, a function of the proportion of cases each physician consideres abnormal (Spitzer & Fleiss, 1974). If two physicians each consider half the cases abnormal, they will agree 25% of the time by chance alone. If they each consider 80% of the cases abnormal, they will agree 64% of the time by chance alone. Hence, since the proportion of abnormal cases varies across studies, the level of agreement by chance varies across studies. Thus, an overall agreement rate of 80% in one study may be 55% more than chance expectation, whereas in a second study it is only 16% more than chance expectation (Spitzer & Fleiss, 1974).

This phenomenon is reflected in the current study, in which only hospitalised patients were examined, with more than 40% of the patients having ECOG score 2 or worse, and 21% to 31% of cases having ECOG score 3 or 4. This

**Table V** Simplified performance status assessment among 100 cancer patients by three observers

| *ECOG score* | *Agreement (proportion of cases with agreement among all three observers)* |
|---|---|
| 0–2 | 0.92 |
| 3–4 | 0.82 |

resulted in an overall agreement of 40% among three observers. In contrast, Conill *et al.* (Conill *et al.*, 1990) examined a patient population with a lower frequency of severe abnormalities, as 79% to 81% of their patients had ECOG Performance Status 0 or 1, and only 5% to 7% had PS 3 or 4. There was a coincidence degree of 59% in the rating according to ECOG Performance Status by the two observers. Contributing to the difference in agreement rates between the present study (40% agreement) and the study by Conill *et al.* (59%) is also the different number of observers, as the more observers, the lower the inter-observer agreement rate would be expected to be (Segall, 1960; Conn & Spencer, 1972).

The order in which the observers interviewed patients for assessment of performance status may theoretically introduce systematic biases if the order is not completely randomized. In the current study, observer 1 had universally performed the first interview with patient, while the order of interview by observer 2 and 3 was random. However, the similar distribution of patients in the respective performance status classes achieved by the three observers as shown in Table III does not point towards any systematic bias in the results.

Performance status has in recent years become a tool in the growing field of psychometric testing of scales for measuring quality of life among cancer patients. In the process of developing such scales the validity is often tested by comparing the results from rating on the quality of life scale to the PS assessment, e.g. as described by Schipper *et al.* (Schipper *et al.*, 1984) in the evaluation of the Functional Living Index – Cancer. This new use of the PS assessment obviously necessitates a solid knowledge of the validity and the reliability of the PS scale itself.

While PS assessment has traditionally been performed by physicians, this is not necessarily the case in scales measuring quality of life. Slevin *et al.* conclude from a questionnaire study that a reliable and consistent method of measuring quality of life in cancer patients must come from the patients themselves (Slevin *et al.*, 1988). Given this difference with respect to observers of the scale, together with the inter-observers variability in PS assessment, one should not expect correlations between quality of life scales and PS to be very high, even if the former scales were valid.

In conclusion, the reliability of the ECOG Scale of Performance Status, as evaluated by the degree of inter-observer variability, is fair, offering additional support for its widespread use, for example as a prognostic factor or as an inclusion criterium for entry into clinical trials. Agreement is higher in patients with good performance status than in more affected patients, a fact which must be born in mind in the interpretation of data from trials dealing with this topic.

### References

COHEN, J. (1960). A coefficient of agreement for nominal scales. *Educat. & Psychol. Measurement*, **XX**, 37–46.

CONILL, C., VERGER, E. & SALAMERO, M. (1990). Performance status assessment in cancer patients. *Cancer*, **65**, 1864–1866.

CONN, H.O. & SPENCER, R.P. (1972). Observer error in liver scans. *Gastroenterology*, **62**, 1085–1090.

FLEISS, J.L., NEE, J.C.M. & LANDIS, J.R. (1979). Large sample variance of kappa in the case of different sets of rates. *Psychol. Bull.*, **86**, 974–977.

GANZ, P.A., HASKELL, C.M., FIGLIN, R.A., LA SOTO, N. & SIAN, J. (1988). Estimating the quality of life in a clinical trial of patients with metastatic lung cancer using the Karnofsky performance status and the functional living index – cancer. *Cancer*, **61**, 849–856.

HUTCHINSON, T.A., BOYD, N.F. & FEINSTEIN, A.R. (1979). Scientific problems in clinical scale as demonstrated in Karnofsky index of performance status. *J. Chronic. Cis.*, **32**, 661–666.

KARNOFSKY, D.A., ABLEMAN, W.H., CRAVER, L.F. & BURCHENAL, J.H. (1948). The use of nitrogen mustard in the palliative treatment of carcinoma. *Cancer*, **1**, 634–656.

KORAN, L.M. (1975). The reliability of clinical methods, data, and judgments. *N. Eng. J. Med.*, **293**, 642–646.

LUND, B., WILLIAMSON, P., VAN HOUWELINGEN, H.C. & NEIJT, J.P. (1990). Comparison of the predictive power of different prognostic indices for overall survival in patients with advanced ovarian carcinoma. *Cancer Res.*, **50**, 4626–4629.

MOR, V., LALIBERTE, L., MORRIS, J.N. & WIEMANN, M. (1984). The Karnofsky performance status scale: an examination of its reliability and validity in a research setting. *Cancer*, **53**, 2002–2007.

SCHAG, C.C., HEINRICH, R.L. & GANZ, P.A. (1984). Karnofsky performance status revisited: reliability, validity and guidelines. *J. Clin. Oncol.*, **2**, 187–193.

SCHIPPER, H., CLINCH, J., MCMURRAY, A. & LEVITT, M. (1984). Measuring the quality of life of cancer patients: The Functional Living Index – Cancer: development and validation. *J. Clin. Oncol.*, **2**, 472–483.

SEGALL, H.N. (1960). The electrocardiogram and its interpretation in a study of reports by 20 physicians on a set of 100 electrocardiograms. *Can. Med. Assoc. J.*, **82**, 2–6.

SLEVIN, M.L., PLANT, H., LYNCH, D., DRINKWATER, J. & GREGORY, W.M. (1988). Who should measure quality of life, the doctor or the patient? *Br. J. Cancer*, **57**, 109–112.

SPITZER, R.L. & FLEISS, J.L. (1974). A re-analysis of the reliability of psychiatric diagnosis. *Br. J. Psychiatry*, **125**, 341–347.

SWENERTON, K.D., LEGHA, S.S., SMITH, T., HORTOBAGYI, G.N., GEHAN, E.A., YAP, H.Y., GUTTERMAN, J.U. & BLUMENSCHEIN, G.R. (1979). Prognostic factors in metastatic breast cancer treated with combination therapy. *Cancer Res.*, **39**, 1552–1562.

SØRENSEN, J.B., BADSBERG, J.H. & OLSEN, J. (1989). Prognostic factors in advanced adenocarcinoma of the lung. A multivariate regression analysis of 259 consecutive patients. *Cancer Res.*, **49**, 5747–5754.

WOOD, C.A., ANDERSON, J. & YATES, J.W. (1981). Physical function assessment in patients with advanced cancer. *Med. Pediatr. Oncol.*, **9**, 129–132.

YATES, J.W., CHALMER, B. & MCKEGNEY, F.P. (1980). Evaluation of patients with advanced cancer using the Karnofsky performance status. *Cancer*, **45**, 2220–2224.

ZUBROD, C.G., SCHEIDERMAN, M., FREI, E., BRINDLEY, C., GOLD, L.G., SHNIDER, B., OVIEDO, R., GORMAN, J., JONES, R., JONSSON, U., COLSKY, J., CHALMERS, T., FERGUSON, B., DEDERICH, M., HOLLAND, J., SELAWRY, O., REGELSON, W., LASAGNA, L. & OWENS, A.H. (1960). Cancer – appraisal of methods for the study of chemotherapy of cancer in man: thiophosphamide. *J. Chronic. Dis.*, **11**, 7–33.

ØSTERLIND, K. & ANDERSEN, P.K. (1986). Prognostic factors in small cell lung cancer. Multivariate model based on 778 patients treated with chemotherapy with or without irradiation. *Cancer Res.*, **46**, 4189–4194.